# How to effectively control vertical collusion in bidding for government investment projects-Based on fsQCA method

**DOI:** 10.1371/journal.pone.0274002

**Published:** 2022-09-13

**Authors:** Chongsen Ma, Yun Chen, Wenxi Zhu, Liang Ou

**Affiliations:** School of Traffic & Transportation Engineering, Changsha University of Science and Technology, Hu Nan, China; Sichuan University, CHINA

## Abstract

The impact of collusion during the bidding processes of Chinese government investment projects is a major concern in academic and policy circles, as collusion breeds corruption and destroys the credibility of governments. Furthermore, it negatively impacts successful project completion, leading to cost overruns and the illegitimate enrichment of colluding agents, regardless of the intended social benefits. Using data from 166 selected regional policy implementations as the research sample, this paper uses the fuzzy set qualitative comparative analysis method to conduct a group analysis of typical cases. The purpose of this study is to identify and better understand the cooperative regional policy implementation environments in China and to identify effective methods to improve the governance quality of collusion controls in construction investment project bidding processes. Five key control paths are identified, covering 94% of the cases. It is also found that in lower social collusion situations, reasonable market competition regulations can directly reduce collusive behavior. The research results will help the government to formulate more adaptive control policies and promote high-quality development of government investment projects.

## 1. Introduction

In the domain of engineering construction in China, collusion in the bidding process for government investment projects is forbidden, as doing so breeds corruption and destroys the credibility of the government. In Chinese public projects, collusive behavior includes typical forms such as assisting in the formation of bid-rigging alliances, internalizing bidders, leaking bids, inducing traditional bid evaluation experts, lowering minimum standards, and collusive practices resulting from multiple collaborations. At the same time, due to the natural information asymmetry in the bidding process of government investment projects. In the case of information asymmetry, the bidding participant with more information will gain a greater advantage. Specifically, in the case of vertical collusion, the government side has an inherent information advantage in government investment projects. Therefore, firms that choose to engage in collusion have a greater chance of gaining excess revenue. Furthermore, it negatively impacts successful project completion, leading to cost overruns and the illegitimate enrichment of colluding agents, regardless of the intended social benefits. Nevertheless, collusion continues. Therefore, the development of effective measures to control collusion during the bidding process of government investment projects in China has become a crucial issue.

Substantial research into this and similar issues has been accomplished in Western economies, focusing on the emergence of collusion and corruption. Delphi or game theory methods are commonplace, and the examination of internal connectivity between cases is less examined. Notably, the differences of cultural and governmental differences between China and the West render much of the international research non-generalizable to domestic problems. Furthermore, according the findings of scholars in several fields, policy formulation in China must fully consider the domestic regional cultural and economic differences. Thus far, Chinese research on government investment project bidding collusion has focused on the gaming interests of participants, the identification of collusive behaviors, quotation interval measurements, and prevention methods. The integration of specific regional concerns has not been investigated.

Bidding collusion has been divided into vertical and horizontal types, where horizontal collusion occurs at the same hierarchical level among conspirators. In vertical collusion, there are hierarchical differences between conspirators at upper and lower levels. Additionally, owing to the information asymmetry of the different levels, actors with a disadvantage have an *information demand*, whereas actors with an information advantage have an *information rent-seeking supply*. Hence, they engage in information trading. In the absence of additional costs, collusion is likely to occur between project bidding stakeholders regardless of information asymmetry, and the possibility of collusion is greater when the costs are symmetric. However, in the presence of additional costs, asymmetric costs of collusion become apparent. Hence, vertical collusion is more complex, and it is expected that the attractiveness of collusion will differ, depending on the region in China.

Through the summary of the existing research, we can find that the following two problems are still waiting to be solved in the current research:

1. the existing research lacks case-based research on bidding vertical collusion control strategy
2. how to combine the control strategy with the external environment of the controlled area to make the control strategy more adaptable

This paper examines the quality of vertical collusion controls among 166 regional policy implementation cases in China using the fuzzy set qualitative comparative analysis (fsQCA) method to provide theoretical and practical guidance for seeking reasonable control paths. This article adds to the literature by extending the application of fsQCA to vertical collusion in Chinese government project bidding processes, introducing regional propensity for social collusion as a factor, and proposing five effective control paths for collusion controls.

The research objectives of this paper are mainly reflected in the following two points:

- Building a case-based control strategy for vertical collusion in tendering
- Take the external social environment into account in the strategy construction process to enhance the adaptability of the strategy

## 2. Literature review

### 2.1. Tender collusion related research

Lee was the first to study collusion in engineering cases in China, concluding that it leads to a reduction in the number of effective bidders. Furthermore, it indirectly leads to project failure [1]. Charles et al. showed that the existence of collusion is more insidious [2]. Bowen’s research pointed out that in developing countries, contractors adopt collusive behaviors when competing for projects [3]. The research of Chinese scholars mainly focused on the irregular behaviors of agents. Guo Nanyun et al. considered collusion to be a collaborative behavior among project participants designed to obtain profits above those identified by the principles of fair bidding and competition [4]. Although there are many government investment projects designed to improve the social good, corresponding regulatory and statutory measures are lacking, providing space for the exploitation of processes for personal gain. Horizontal and vertical collusion can take place between bidders, agents, and combinations of both, and doing so relies on the existence of information asymmetry and negative externalities. Vertical collusion has a particularly damaging effect on the fair market environment. Zhu Shun asserted that the supervision and control of such paradigms could be improved by strengthening legislation [5]. Zhang used the D-S evidence synthesis method to construct a comprehensive judgment matrix for group decision-making, and finally get the green procurement evaluation index system for government public projects [6].

### 2.2. Research related to the generation of vertical collusion

Principal–agent and game theories are the main methods used by Chinese scholars when analyzing the causative factors of engineering collusion. Huang Wenjie pointed out that, although collusion is induced by excessive competitive pressures, the root cause is fact that the potential returns far outweigh the punishment. Yu Xiaozhong showed that the information asymmetry caused by multiple entrustment relationships in government investment construction projects and the low costs of collusion are the main reasons for its emergence [7]. Cheng Shuping et al. indicated that high profits from bidding maneuvering are the main inducements of vertical collusion [8]. Jeanine et al. demonstrated that, although collusion is possible without additional costs, they promote its occurrence [9]. Chen et al. found that technical and environmental causes are more indicative of the emergence of vertical collusion [10]. Ding Zhang argued that collusion does not arise from the influence of a single factor; it emerges from their combination.

### 2.3. Detection and determination of collusion phenomenon

Detecting and verifying collusion is a prerequisite for managing the problem. Patrick et al. constructed a model for identifying bidder collusion signaling [11]. Chotibhongs et al. built an empirical model for determining the existence of collusion using historical data [12]. Later, a prediction model for detecting the possibility of bidder collusion was designed on this basis [13]. Yin Yilin et al. used a hierarchical analysis of relevant phenomena to provide a method for determining the existence of collusion [14]. Chen et al. developed a mathematical collusion identification model that accounts for multi-interest engineering bidding processes [15]. Li Lihong et al. identified 17 factors influencing the efficacy of bidding supervision [16]. Li et al. indicated unbalanced bidding can increase the owner’s cost and decreases the fairness of the competitive bidding process, and constructed an extended TOPSIS-based model for the identification of unbalanced bidding. His research can help the owners can undertake objective decision-making to identify and prevent unbalanced bidding at the stage of procurement [17].

Scholars have provided diverse solution paths to controlling collusion in bidding for government investment projects. Wu et al. combined prospect and game theories to establish a collusion deterrence model for government investment projects [18]. Wang et al. constructed a control model to prevent collusion between agents and bidders based on the”prisoner’s dilemma” game [19]. Chen et al. analyzed collusion conditions using a cooperative game model, which provides a reference for collusion control policy formulation [20]. Hengquan Zhang et al. constructed a two-sided game model between owners and bid evaluation experts, resulting in a control model that prevents collusion between bid evaluation experts [21]. Liu et al. believe that fraud in the operation of construction companies can be effectively solved through supply chain finance measures based on blockchain technology, and has constructed a hybrid chain based on PANDA (a consensus algorithm based on the public chain) and X-Alliance (a consensus algorithm based on alliance chain), which can assist in trade authenticity review and transaction risk assessment. The hybrid chain based on PANDA (a consensus algorithm based on the public chain) and X-Alliance (a consensus algorithm based on the alliance chain) can help enterprises in trade authenticity review and transaction risk assessment [22]. His research provides a new solution to the control of collusion in tendering. Liu and Shi indicated that BIM technology has become an indispensable tool for the construction industry [23]. However, the current application of BIM technology combined with bidding vertical collusion control is still relatively small and deserves further in-depth study.

### 2.4 Limitations of existing studies

It can be learned from the current literature that vertical collusion is abundant in the bidding of Chinese government investment projects. However, extant results are limited in that they mainly focus on game and principal–agent theories, which concern themselves predominantly with the influence and relationships of agents. Studies are scant on the effects of factorial combinations of vertical collusion factors in Chinese government investment bidding. Fewer papers have studied real vertical collusion case data, and there is a lack of successful control path studies overall.

## 3. Research and methods

### 3.1. Selection of research method

Qualitative comparative analysis (QCA) is a case-based research method that combines qualitative and quantitative approaches. Compared with traditional research methods, QCA reflects the non-linear relationships between variables and outcomes by examining the multiple influences of social phenomena. It is mainly applicable to small sample cases. Modern QCA methods include fsQCA, crisp set QCA (csQCA), and multi-value QCA (mvQCA). The csQCA method treats full case membership as a binary variable (zero or one), which helps overcome subjectivity to a certain extent. The mvQCA method accounts for multinomial categorical case data. Although many collusion control policies have been advocated in China, relevant laws and regulations are difficult to find. Furthermore, it is difficult for governments beneath the municipal level to obtain relevant data to either detect or deter collusion. To solve the problem of this research, fsQCA is best, as it allows varying degrees of case membership based on affiliation, which is assigned using the relative position between indicators.

**Fig 1:**
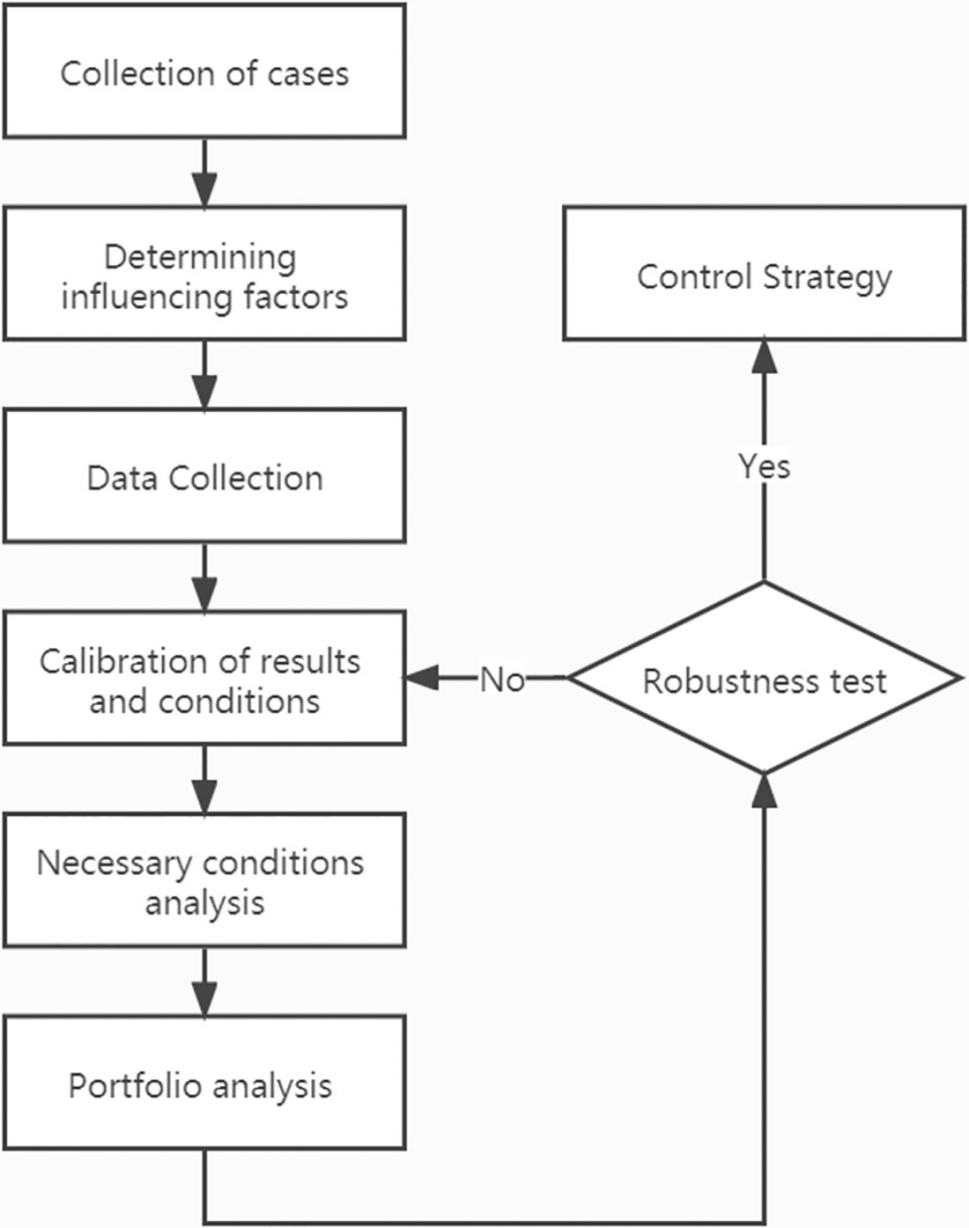
Technology roadmap.

Therefore, the fsQCA method is leveraged to analyze the factors influencing collusion control at different time periods and in different environments. The factors arising from vertical collusion are more diverse than horizontal collusion, and the efficacy of control is influenced by a variety of complex factors. Similarly, the factors change and interact to wildly affect the dependent results in a *fuzzy* fashion. The technology roadmap of the research is shown in Fig 1.

### 3.2. Case selection for research

Accurate and adequate case selection has a significant impact on the reliability of study results, especially with fsQCA. The paper judges the efficacy of collusion controls in terms of their applicability to the prevailing external environment based on the number of changes in administrative cases in the 2 years before and after the changes in relevant management tools and external environment were made.

The criteria for selecting cases were as follows. First, to guarantee the reliability of case indicators, public data were collected from the official National Bureau of Statistics website, various Chinese General Social Survey datasets, and the official Judgment Document Network website, which ensures source authenticity and reliability. Second, to ensure the diversity of case sources, those from 31 provinces, municipalities, and autonomous regions across China were collected to ensure data diversity. The sample cases included those with and without effective collusion controls between 2007 and 2021. Third, to ensure the representability of cases, after initial data collation, cases lacking new policies and control measures were discarded to avoid redundancy and bias. Similarly, cases containing obscure indicators or those with differences depending on their source were eliminated. 166 cases were finally included.

### 3.3. Variable selection and assignment

To construct an evaluation index for the causal factors of collusion, the latent Direchlet allocation (LDA) topic model was applied. LDA is commonly used unsupervised Bayesian machine learning algorithm that automatically extracts topics from large-scale texts without manual processing. By counting the input text, the numbers of words in each of *M* documents are recorded. The distribution of topics and words is then found for each document. Specifically, LDA is used in this research to identify the topical semantics of 100 highly cited papers and 40 judgments from collusion cases from 2006 to 2021. Highly frequency words were selected for factor analysis, and Gensim and Numpy libraries were used to construct the LDA model after integrating a specialized thesaurus and deactivating undesirable terms.

In this research, keywords “government investment projects” and “collusion” were used to search the China National Knowledge Infrastructure database and 100 core papers with high application rates from 2000 to 2021. A total of 21 case judgments were selected as the initial data source, and after manual screening, 43 paper items were retained.

**Fig 2:**
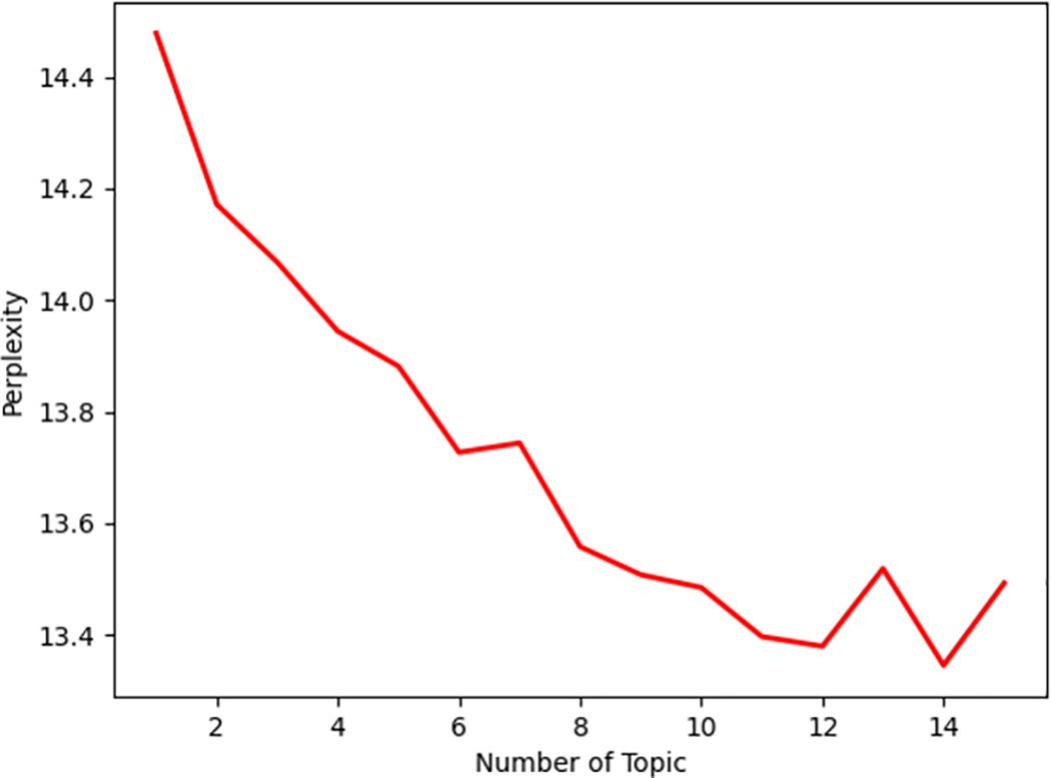
Topic perplexity of the LDA model.

Relevant vertical collusion evaluation and influence indicators were obtained from both Chinese and international literature, and the key indicators were refined using interviews. The model perplexity of the analyzed texts is illustrated in Fig 2, where it can be seen that the articles had the least confusion when 10 topics were selected. But the best results were obtained using nine topics (see Table 1). The probability of relevant factors being generated has a small impact on the study, and after referring to the subject terms, they were statistically collated, and interviews were conducted with relevant experts. The highest impacting indicators of collusion are thus shown in Table 2.

**Table 1:** Analysis results of high-frequency subject words in the LDA model.

**Topic0**		**Topic1**		**Topic2**		**Topic3**	
Word	Prob	Word	Prob	Word	Prob	Word	Prob
Assessment	0.029	Engineering	0.014	Quotation	0.008	Relationship	0.015
Land	0.017	Contract	0.013	House	0.004	Land	0.014
Performance	0.012	Debt	0.011	Resettlement	0.004	Corruption	0.013
Hantec	0.010	Original trial	0.007	Agreement	0.004	Second Party	0.012
Level	0.010	Reached	0.007	Mining	0.004	Agreement	0.009
···		···		···		···	
**Topic4**		**Topic5**		**Topic6**		**Topic7**	
Word	Prob	Word	Prob	Word	Prob	Word	Prob
Collusion	0.011	Government	0.030	Bid evaluation	0.069	Government	0.035
Contractor	0.011	Local	0.025	Experts	0.045	Collusion	0.021
Profits	0.009	Economy	0.012	Evaluation	0.014	Society	0.014
Opportunism	0.009	Capital	0.010	Strategy	0.014	Organization	0.012
Public	0.007	Constraints	0.010	Bidders	0.011	Relationship	0.009
···		···		···		···	
**Topic8**							
Word	Prob						
Government	0.023						
Local	0.015						
Collusion	0.009						
Incentives	0.007						
Central	0.007						
···							

**Table 2:** Key influencing factors for vertical collusion in bidding.

Dimension	Influencing Factor	Representative Scholar
Socially complicit atmosphere	Degree of social collusion tendency	Wang Jiaming [24], hee-chang [25], Wachs [26]
	Degree of government collusion tendency	Zhang B [27], Ernest E [28], Bauhr M [29]
Degree of government fairness	Degree of judicial impartiality	Vadlamannati [30], Anders G [31]
	Degree of trial impartiality	Kang Liangzhao [32], Dongmei Feng [33]
	Efficacy of supervision procedures	Dong Ye [34], Zeng Linqi [35]
Market Tightness	Degree of market competition	Sun Wangming [36], Kapoguzov [37]
Local economic development	State of economic development	Mukherjee [38], Fang Yuan [39]
Control efforts	Existence of special actions and laws in the given year	Sun Wangming [36], Dong Ye [34]
Quality of control	Percentage decrease in the growth rate of collusion cases	

The data selected for this paper were obtained from the Chinese General Social Survey and the Chinese National Statistical Yearbook. Among them, the Chinese General Social Survey (CGSS) of China Survey and Data Center is the earliest national, comprehensive, and continuous academic survey project for China, which selects more than 10,000 households in all provinces and autonomous regions of mainland China for continuous cross-sectional surveys. The database is therefore highly credible.

The finalized key influencing factors include the *degree of social collusion tendency* (CGSS2017 a59h, S41), the *degree of government collusion tendency* (f241–f245), the *degree of judicial impartiality* (CGSS2015 f61–f63), the *degree of trial impartiality* (f71–f76), the *efficacy of supervision procedures* (f161–f174), the *degree of market competition*, the *state of economic development*(China National Statistical Yearbook), and the *existence of special actions and laws in the given year*. The relevant situation of each province was clarified separately. The dependent variable is *quality of control*.

When using the fsQCA method, each condition and outcome are considered a set, and each case has an affiliation score in the set. The tool was calibrated for variable data according to the level of affiliation of cases with the characteristic variables, and all data were assigned values between [0, 1] with larger values indicating higher affiliation (apart from control strength). Variables were then assigned based on whether the initial value was greater than the second quartile of all values. If so, it was changed to “1;” otherwise “0.” Control strength was assessed according to whether there was a special control in the given year. The variables were interpreted and categorized as shown in Table 3.

**Table 3:** Interpretation of vertical collusion control quality variables.

Category	Name	Explanation	Data source
Result Variables	Quality of control (Result)	Percentage decrease in the growth rate of collusion cases	Judgment Documents Network
Conditional Variables	Socially complicit atmosphere (hmqxd)	Acceptance of collusive behavior by the various participants in the conspiracy	CGSS2017
	Degree of government fairness (zfljcd)	The degree of perceived social justice in the government’s implementation of relevant policies	CGSS2015
	Market Tightness (scjjcd)	The intensity of competition among construction companies	Reflected by the number of private construction enterprises and the total construction industry output value
	Local economic development (gdp)	Local economic development	GDP
	Control efforts (zxld)	For the enforcement of relevant regulations	Website of the General Office of the CPC Central Committee

This method converts the data into fuzzy set affiliation scores using direct calibration of antecedent conditions and outcomes based on extant theory (Table 4).

**Table 4:** Calibration of results and conditions.

Result and condition codes	Calibration		
	Fully subordinate	Intersections	Completely unaffiliated
Result	>0	-	<0
hmqxd	7.7	-	0
zfljcd	9.69	-	0
scjjcd	230.625	-	0
gdp	41398.45	-	0
zxld	1	0.67	0

Regarding the evaluation of quality of control (Result, Table 4), the increase in the number of collusion violations (i.e., proportional change in velocity) was used, where a positive rate of increase indicates that the controls did not have the desired effect. A negative rate means that the controls had the desired effect. The ratio is a continuous variable modified such that a positive rate was set to zero, and cases with a negative rate were set to one.

The condition code, hmqxd, refers to the socially complicit atmosphere. Drawing on existing research, this paper applied two variables, degree of social collusion tendency and the degree of government collusion tendency, as descriptors. Data from 31 provinces and municipalities were used alongside questionnaire data based on a five-point ordinal scale. Variables were assigned values based on whether the initial value was greater than the second quartile of all values. If greater, then one; otherwise zero. A value of one represents a local propensity for collusion and a worse external environment for governance than 50% of all cases.

The condition code, zfljcd, refers to the degree of government fairness. Drawing on existing research and advance data from the LDA exercise, a five-point scale was used to measure the degree of judicial impartiality, the degree of trial impartiality, and the efficacy of supervision procedures using data from the China Social Survey. Variables were assigned values based on whether the initial value was greater than the second quartile of all values. If greater, then one; otherwise zero. A value of one represents the local propensity for collusion and a worse external environment for governance than 75% of the cases.

The condition code, scjjcd, refers to market tightness (competitiveness). This factor has a large impact on whether firms choose to collude. Therefore, the degree of market competition was selected for analysis. The source of the value is the ratio of the total construction output to the number of construction firms. If the value for a case is greater than 50% of all cases, then the local market is considered to be less competitive, and firms have access to a relatively sufficient number of projects, in which case a value of one is assigned; otherwise, a value of zero is assigned.

The condition code, gdp, refers to the local state of economic development per year. Variables were assigned values based on whether the initial value was greater than the second quartile of all values. If greater, then one; otherwise zero.

The condition code, zxld, refers to control efforts, implying the existence of special actions and laws in the given year. A value was assigned based on the year in which the relevant legislation or special measure was introduced to remedy the problem.

### 3.4. Measurement and calibration

Based on the above variable criteria and meaning interpretations, the cases were assigned values, and a final truth table excerpt is shown in Table 5.

**Table 5:** Calibrated vertical collusion control truth table (excerpt).

ID	hmqxd	zfljcd	scjzcd	gdp	zxxd	result
1	0	0	1	0	0	0
2	0	0	0	0	0	1
3	1	1	1	0	0	1
4	1	1	1	0	0	1
5	1	1	1	0	0	0
6	1	1	0	0	0.6	0
7	0	0	0	0	0	1
8	0	0	0	0	0	1
9	1	1	0	1	0.6	0
10	1	1	1	0	0	1
11	1	1	1	0	0	1
12	1	1	1	0	0	1
13	1	1	1	0	0	1
14	1	1	1	0	0	1
····						

## 4. Result

### 4.1. Necessary conditions analysis

Before analyzing the relevant groupings, it is necessary to determine whether a single factor may have affected the variables, changing the quality measure of control. In fsQCA, a necessary condition is one that affects the outcome variable. Generally, one is considered necessary if the consistency is greater than 0.9 [40]. Thus, necessity was analyzed using fsQCA, and the results are shown in Table 6, revealing that the consistency of all conditions was less than 0.9, meaning that no condition led to the inevitable occurrence of a result, all influencing factors were retained, and the data can be further analyzed.

**Table 6:** Necessity test form.

Variables	Consistency	Coverage
hmqxd	0.193333	0.828571
~hmqxd	0.806667	0.923664
zfljcd	0.213333	0.800000
~zfljcd	0.786667	0.936508
scjjcd	0.473333	0.865854
~scjjcd	0.526667	0.940476
gdp	0.253333	0.904762
~gdp	0.746667	0.903226
zxld	0.220000	0.820895
~zxld	0.780000	0.930048

Note:“~”absence.

### 4.2. Portfolio analysis of influencing factors

Based on a related study by Ragin, 0.8 and 1 were set as the consistency and case thresholds [41,42]. The results were analyzed by outputting complex, streamlined, and intermediate solutions. Generally, an intermediate solution outperforms complex and streamlined solutions. The conditions jointly contained in the streamlined and intermediate solutions are called “core conditions,” and those contained only in the streamlined solution are called “auxiliary conditions.” The antecedent condition configurations for collusion case growth rate reduction are shown in Table 7.

**Table 7:** Antecedent condition configuration of the increase in the growth rate of collusion cases.

Variables	P1	P2	P3	P4	P5
hmqxd	⊗	-	⊗	⚫	-
zfljcd	-	-	⊗	⚫	⚫
scjjcd	⊗	⚫	-	⚫	⊗
gdp	-	⊗	-	-	●
zxld	-	⊗	⚫	-	-
Consistency	1	0.948276	0.962963	0.9375	0.925926
Row coverage	0.473333	0.366667	0.173333	0.1	0.033333
Unique coverage	0.34	0.293333	0.053333	0.026666	0.02
Solution consistency	0.969739				
Solution coverage	0.94				

*Note*: “⚫” (presence) and”⊗“(absence) represent core condition,”●”(presence)and “⊗“(absence)represent peripheral conditions.”-”represent no impact.

From Table 7, it can be seen that the total consistency was 0.969739, which meets the requirement; the total coverage was 0.94, indicating that the cases explain 94% of the reasons for vertical collusion. Five different paths that may lead to an increase in the growth rate of collusion (i.e., less than expected quality of control over collusion) cases were also obtained. Path 1 is the most important, with a unique coverage greater than the other paths. The core conditions for this path were ~hmqxd and ~scjjcd, which represent the ability to achieve better control over vertical collusion in regions with a lower degree of collusion propensity and a weaker degree of market competition, even if the government is less impartial and has less control over collusion events. This relationship reflects the importance of the external environment in controlling collusion. It is also necessary to improve the overall quality of society, create a clean social environment, increase education, establish reasonable entry thresholds, avoid disorderly competition in the market, control the degree of competition, and achieve joint control of collusion tendencies.

Path P1 (~hmqxd*~scjjcd) reflects a socially complicit atmosphere × market tightness. This path shows that the effective control of vertical collusion in bidding can be achieved when both the willingness to socially collude and the degree of market competition are low. It also shows that market tightness and the society’s tendency to collude can play a complementary role.

Path P2 (scjjcd*~gdp*~zxld) reflects market tightness × ~local economic development status × control effort. This path shows that, in the case of insufficient government enforcement and a low overall economic level of society, if the bidding of government investment projects is in a highly competitive market situation, effective control of vertical collusion can be achieved, because the collusion brings sufficient expected benefits neither to either enterprises nor government parties.

Path P3 (~hmqxd*~zfljcd*zxld) implies ~ socially complicit atmosphere × ~degree of government fairness × control effort. This path suggests that, with a low propensity for social collusion and a high level of government integrity, and effective government controls, collusion control can be effectively enhanced through the joint regulation of internal control efforts and external environments.

Path P4 (hmqxd*zfljcd*scjjcd) reflects a socially complicit atmosphere × degree of government fairness × market tightness. The path illustrates that, in cities with high social collusion and low government integrity, the probability of vertical collusion can be effectively reduced by increasing the degree of market competition and reducing the arbitrage margin of government investment projects so that their profitability is at the conventional market level.

Path P5 (zfljcd*~scjjcd*gdp*~zxld) reflects the degree of government fairness × ~market tightness × local economic development status × ~control effort. This path indicates that, in the case of low government integrity and a high level of local economic development, if the degree of competition in the market is low, even if government control is insufficient, a better control effect can be achieved.

By examining these five paths, it can be seen that market tightness, the propensity to collude, and the intensity of enforcement are the most important requirements, which further indicates that in an actual control process, the control effect is best for these three indicators.

### 4.3. Robustness test

Adjusting for the level of consistency is a common method of determining QCA robustness (Schneider and Wagemann, 2012). According to Schneider et al., the robustness of a method refers to its capacity to remain unaffected by small variations in the methodological parameters. Hence, it can be ascribed to “reliability.”

In the case of this research, the consistency level threshold was increased to 0.88, and group analysis was performed again using the same frequencies. These results are shown in Table 8. Paths 1, 2, and 4 remained unchanged. Paths 3 and 5 behaved differently, but the interpretation mechanism behind the groupings was the same. The overall consistency and coverage changed to a small extent. Therefore, the findings in this paper were not found to have changed substantially with an increased consistency threshold. Hence, the model is robust and reliable.

**Table 8:** Robustness test form.

Variables	P1	P2	P3	P4	P5
hmqxd	⊗	-	⊗	●	●
zfljcd	-	-	⊗	●	●
scjjcd	⊗	●	-	●	-
gdp	-	⊗	●	-	●
zxld	-	⊗	●	-	⊗
Consistency	1	0.948276	1	0.9375	0.925926
Row coverage	0.473333	0.366667	0.06	0.1	0.033333
Unique coverage	0.426667	0.293333	0.013333	0.013333	0.02
Solution consistency	0.975434				
Solution coverage	0.9				

## 5. Conclusions

Government investment bidding policies in China have made great progress in terms of application areas and methods, but the phenomenon of collusion between governments and enterprises remains a problem, despite its repeated prohibition. Previous studies on vertical collusion in bidding have mostly focused on the classification of collusive behavior, behavioral decisions of bidding participants, and prediction of collusion tendencies. However, the research on vertical collusion control is very limited. Therefore, this study fills this knowledge gap. The main contribution of this paper is the novelty it provides, which is reflected in the incorporation of social collusion tendency into the process of strategy formulation, the use of LDA thematic model into the process of constructing indicators, and the analysis of control cases using the fsQCA method. Prior to the current study, there were no good universal measures in China upon which collusion controls could be built.

In this study, the fsQCA method was used on data culled from 166 governance cases in 31 domestic Chinese regions at different periods and under different conditions. Hence, the effects of each variable on the quality of collusion controls were assessed based on their different groupings. From the results of single factor analysis, it was found that a lower degree of market competition is necessary for improving control quality. From a combination of conditions, it was found that several paths affect the quality of vertical collusion control, and improvements depend on choosing different methods for different situations. Based on the five paths ascertained from the analysis, the following three major suggestions are offered to resolve the problem.

- *Reduce the social collusion climate*. The social collusion environment occupies a very important position in Chinese business and governance. The degree of government fairness and local economic development are closely related to the overall social climate. First, improvements to related indicators should be used to effectively reduce the social collusion atmosphere and create a good bidding environment. Controlling the indicated phenomena is important, but providing preventative educational measures would also help. Furthermore, notable cases should be used for publicity and to create a catalog of bidding restrictions/recommendations. For cities with a poor collusion environment, the government should strengthen its management efforts and try to reduce the tendency of social collusion and destroy the collusion environment through strong and effective control measures (such as increasing the enforcement of policies and implementing regular inspection measures); and build a social environment in which "collusion is not possible, not daring to collude, and not wanting to collude" in phases. The government should build a social environment in which people cannot, dare not, and do not want to collude [43].
- *Play the role of market guidance*. Economic development and the degree of market competition influence the attractiveness of collusion. Under conditions of strong market competition, some enterprises are more likely to collude with government officials to ensure their survival. Unfortunately, this destroys fair competition. The degree of competition in the market clearly affects the choice of enterprises. However, in the case of very low competition, the probability that firms will choose to abandon collusion increases. The tendency is to increase collusion at first, followed by a decrease. Path 1, which covers 47% of all cases, showed that with low social collusion, if the degree of market competition can be reasonably controlled, the deterrence of collusion can be better achieved. Therefore, the government should raise the market entry threshold to avoid disorderly competition. When the market competition is too high, even if the projects are obtained through vertical collusion, they cannot obtain enough profits; while when the market competition is too low, the enterprises can obtain enough projects without collusion, and the necessity of choosing collusion is not enough at this time. Therefore, local governments should take the local market environment and project types into consideration and reasonably regulate the competition level of projects for different project types to control the collusion phenomenon. Furthermore, the government’s guiding role should be fully exerted to realize the synergistic control of internal and external environments [43–45].
- *Strengthen supervision and control*. In the unique market environments of China, the government occupies a key position in controlling collusion. Hence, they should strengthen internal and external supervision and give full play to the role of supervision mechanisms. Additionally, controls should be strengthened and mandated. Furthermore, big data and machine learning methods should be supplied to carry out comprehensive analyses of bidding processes to improve fairness. Penalties should also be increased to improve deterrence. This is would also lead to standardized market management at many different levels. Due to the different administrative capacities of provincial governments across China, consideration should be given to building a bidding management system and payment system based on blockchain technology and BIM technology from the national level. The government should encourage the adoption of blockchain technology and BIM technology in bidding management to enhance the transparency and trustworthiness of the bidding process [6,22].

Owing to the limited access to data and the existence of data residuals, the sample of this paper only included 166 cases, which small. Thus, the statistical results may show unstable or limited results, despite the model’s robustness. Therefore, the generality of the control measures proposed in this paper deserves further study. In future research, multiple testing methods and results analyses should be used to offer better cross-check validity.
